# Factors influencing the need and willingness for presbyopic correction: a cross sectional study from south India

**DOI:** 10.1038/s41598-023-50288-w

**Published:** 2023-12-21

**Authors:** Dhruval A. Khurana, N. Swathi, A. R. Rajalakshmi

**Affiliations:** Department of Ophthalmology, Mahatma Gandhi Medical College and Research Institute, Sri Balaji Vidyapeeth, Pondicherry, India

**Keywords:** Physiology, Health care, Health occupations, Medical research, Risk factors, Signs and symptoms, Optics and photonics, Nutrition disorders, Diseases, Refractive errors

## Abstract

Presbyopia is an age-related physiological phenomenon in which eye gradually losses its ability to accommodate. It is one of the leading causes of visual impairment worldwide, especially in adults above the age of 40. If uncorrected, it can significantly impair a patient's quality of life. This study aims to evaluate the factors which affects patient’s need and willingness to accept presbyopic correction. This cross-sectional analytical study was carried out in a semi urban tertiary care hospital from Jan 2021 to June 2022 among patients aged 40 and above who presented to Outpatient department (OPD). Demographic details, medical history, presenting ocular complaints pertaining to presbyopia, spectacle use and decision regarding using near vision correction were noted. Ocular examination included refraction and ocular biometry. Factors that may have influenced complaints of presbyopia or willingness to accept presbyopic correction were analysed. Three hundred and forty two patients with a mean age of 48.55 ± 6.68 years were included. Of these, 262 (76.61%) patients presented with chief complaints related to presbyopia. Those with higher educational qualification (*p* = 0.031), hypermetropia (*p* = 0.021), shallower AC depth (*p* = 0.028) and on medications for systemic ailments (*p* = 0.01), were more likely to present with chief complaints attributable to presbyopia. Among them, those with higher educational qualifications (*p* = 0.02) and skilled workers were more likely to accept near vision glasses (*p* = 0.02), while those with lower Hb (*p* = 0.01) and myopia (*p* = 0.01) were less likely to accept correction for presbyopia. Among the 80 patients without chief complaints related to presbyopia, 35 (43.75%) were not willing to accept near vision glasses. Those with higher BMI (*p* = 0.04) and hypermetropes (*p* = 0.05) were more willing to accept presbyopic correction. Presbyopia constitutes a significant reason for patients above the age of 40 visiting eye care facility. Multiple socio-economic, systemic and ocular factors influenced both the chief complaints related to presbyopia and willingness to accept presbyopic correction.

## Introduction

Presbyopia is an age-related condition wherein the eye gradually losses the ability to accommodate. Around the age of 40 years, this becomes symptomatic as a decrease in near vision, increased working distance, fatigue from near work, ocular discomfort, headaches, asthenopia and need for brighter light for reading^1,2^. At this time, additional converging lenses would be required to see clearly for near. Presbyopia has a negative impact on the quality of life of an individual by reducing the clarity of near vision and increasing dependency on glasses^3^.

Though correctable with spectacles, non-spectacle modes of managing presbyopia like corneal procedures of monovision LASIK, PRK, CK, PresbyLASIK, and Intra-Cor corneal inlays, IOL implantation, and anterior ciliary sclerotomy are also available. While these procedures reduce the dependence on glasses, they require to be repeated periodically to sustain freedom from near-vision spectacles. Also these procedures establish monovision, preserving one eye for distance while correcting the other for near vision^4–6^.

Despite the available modes of correction, according to WHO (2023), uncorrected presbyopia is the most common cause of near vision impairment worldwide^7^. Various reasons may be contribute to this. Apart from age, factors like educational status, occupation, corrected or uncorrected refractive errors, gender etc. may influence patients’ complaints related to presbyopia^1^. They may be unaware of their difficulty in near work or their lifestyle may be such that they do not perceive the need for near vision correction. Lack of knowledge that the condition is correctable, inability to access eye care or purchase glasses compound this situation^8^. Further, prescribed glasses may be uncomfortable for the patient, compromising compliance^3^. This study aimed to examine patients' perceptions and the various factors influencing the necessity and acceptance of presbyopic correction.

## Materials and methods

This cross-sectional analytical study was conducted in a tertiary care hospital in South India between January 2021 and June 2022 among patients aged 40 and above, visiting the ophthalmology outpatient clinic. After obtaining approval from Institute Human Ethics Committee (01/2020/46/IHEC/284), willing patients fulfilling the study criteria were enrolled in the study by consecutive convenient sampling. (Written informed consent was obtained from all the patients included in this study). This study was conducted in accordance to declaration of tenets of Helsinki. Based on the prevalence rate of uncorrected presbyopia (33%) in India and absolute precision of 0.05, minimum sample size was calculated to be 340 patients^9^. Patients with acute or painful ocular conditions, structural and functional abnormalities of anterior and posterior segment which preclude achievement of N6 by spectacle correction and uncontrolled diabetes mellitus (HbA1C > 7.6 or if deemed poor control by treating physician) were excluded.

From all patients, chief complaints and detailed history including past history of spectacle usage, systemic diseases, addictions, long term medications (including medications for diabetes, hypertension, hyper-/hypo- thyroidism and psychiatric medications), ocular trauma and ocular surgeries were elicited. Complaints of difficulty in near vison, eye strain or fatigue if present were considered to be related to presbyopia. Ophthalmic examination included Visual acuity (VA) for distance (using illuminated Snellen’s chart and measured at a distance of 6 m) and near (using Snellen’s near vision chart at a distance of 33 cm) and refractive correction for both, intraocular pressure (IOP) measurement by non-contact tonometer (Topcon CT-800,Topcon Medical Systems, Paramus, New-Jersey, USA), axial length (AXL), anterior chamber depth (ACD) and lens thickness (LT) measurements by A-scan (Appasamy MAX, ophthalmic ultrasound scanner, Appasamy Associates, Chennai, India), keratometry (K1/K2) by automated refractometer (URK-800, Auto Ref/Keratometer, UNICOS Co. Ltd., Daejeon, Republic Of Korea ) as well as amplitude of accommodation (AA) and near point of accommodation (NPA) by Royal Air Force (RAF) ruler. NPA (in meters) was measured uniocularly. AA was calculated as the reciprocal of NPA.

Procedure to measure NPA: Cheek rest of RAF ruler was placed on the patient’s cheek and NPA was measured by push up method^10^.

Push up method—Patient was instructed to look at the target (reduced Snellen’s chart) on RAF ruler. The target was gradually moved close to the patient’s eye till he complained of blurring. The point at which patient first complained of blurring was noted as NPA.

Push down method—Target of the RAF ruler is initially placed close to the patient’s eye and gradually moved away. The distance at which the patient first appreciates clear image of the target is noted as NPA.

If accommodation of the patient was so low that NPA lay beyond the scale, suitable convex lenses were added and NPA was brought within the scale. Dioptric power of the lens which was used was then reduced from the calculated accommodation.

Systemic investigations included random blood sugar (RBS), haemoglobin % and body mass index (BMI)^1^. Patients were asked if they would be willing to accept near vision glasses in their prescription and their response was noted as “yes” or “no”.

As presbyopia is a bilateral condition, the right eye of all patients was used for analysis. Data was entered in MS excel and statistical analysis was performed using Microsoft excel (Version 2022) (Microsoft Corporation, Redmond, WA, USA) & Statistical package for the Social Sciences (SPSS) for Windows, (version 16.0, SPSS Inc., Chicago, III., USA). Privacy and confidentiality of participants was maintained throughout the study.

## Results

Of the 342 patients included in the study, 165(48.24%) were men and 177 (51.76%) were women. The mean age of the study population was 48.55 ± 6.68 years and ranged from 40 to 80 years. Forty four (12%) participants were illiterate, 85 (24.85%) graduates and only 14 (4.09%) had received post graduate education. One hundred and sixty seven (48.83%) participants were skilled workers and 49 (14.32%) were professionals. Ninety-nine (28.94%) participants had DM and 67 (19.59%) were hypertensive. One hundred and fourteen (33.33%) patients were on regular long-term medications. Patients travelled a mean distance of 37.61 ± 38.57 km (range: 1–300) in order to reach this eye care facility. Two-hundred sixty-two (77%) participants presented with chief complaints attributable to presbyopia. Only 82 (23.97%) participants had been previously corrected for near vision.

The mean presenting VA for distance was 6/9 ranging from 6/6 to 6/60. Of these, 314 (9.8%) had no or mild visual impairment, & 28(8.2%) had moderate visual impairment according to ICD-11. Mean presenting near vision was N10 ranging from N 6 to N36. Two hundred and twenty one (64.61) patients were emmetropic, 45 (13.16%) myopic and 76 (22.22%) hyperopic. Among the 121 patients who required correction for distance vision, the refraction ranged from -3.25 D to + 3 D. The additional near vision correction required ranged from nil correction to + 3 D. The mean recorded IOP was 15.09 ± 2.30 mm Hg, AC depth was 3.72 ± 1.03 mm, Lens thickness was 3.29 ± 0.87 mm, K1 was 44.41 ± 1.36 dioptre, K2 was 44.85 ± 1.30 dioptre, AA was 2.41 ± 1.17 cm, NPA was 49.79 ± 35.10 cm, NPC was 9.53 ± 1.26 cm and axial length was 23.23 ± 10.99 mm ranging from 20.05 to 24.97 mm.

Based on the complaints of presbyopia, participants were divided into 2 groups—those who had complaints related to presbyopia (*n* = 262) and those who did not (*n* = 80). On comparing the two groups, it was found that those with DM (*p* = 0.013), HTN (*p* = 0.007), high RBS value (*p* = 0.008), requirement for chronic medication (*p* = 0.011), higher education qualification (*p* = 0.031) and hypermetropia (*p* = 0.021) were more likely to present with chief complaints attributable to presbyopia. In addition, it was found that those with complaints of presbyopia had a lower AC depth than those who did not had complains of presbyopia (*p* = 0.028). **(**Tables 1 and 2) A regression analysis model constructed using these parameters showed only educational qualification to be statistically significant with regards to complaints of presbyopia. (R square = 0.07, *p* = 0.01).

**Table 1:** Comparison of socio-demographic parameters between patients with and without complaints related to presbyopia.

	Patients with complaints related to presbyopia (*n* = 262)	Patients without complaints related to presbyopia (*n* = 80)	*p* value
Age (years)			0.062
(mean ± SD)	48.18 ± 6.57	49.78 ± 6.95	
Gender n (%)			0.102
Male	120 (45.8%)	45 (56.2%)	
Female	142 (54.2%)	35 (43.8%)	
Educational qualification *n* (%)			**0.031**
Illiterate	28 (10.7%)	16 (20.0%)	
Primary School	72 (27.5%)	28 (35.0%)	
Secondary School	77 (29.4%)	22 (27.5%)	
Graduate and above	86 (32.2%)	33 (42.3%)	
Occupation n (%)			0.259
Skilled and above	177 (67.6%)	66 (82.5%)	
Semiskilled	74 (28.2%)	11 (13.8%)	
Unskilled	11(4.2%)	3 (3.8%)	

Significant values are in [bold].

**Table 2:** Comparison of ocular and systemic parameters between patients with and without complaints related to presbyopia.

Systemic Parameters, n (%)	Patients with complaints related to presbyopia (*n* = 262)	Patients without complaints related to presbyopia (*n* = 80)	*p* value
Hypertensives	43 (16.4%)	24 (30.0%)	**0.007**
Diabetics	67 (25.6%)	32 (40.0%)	**0.013**
Patients taking chronic medications	78 (29.8%)	36 (45.0%)	**0.011**
BMI (kg/m^2^)			0.943
Under Weight (< 18.5)	6 (2.3%)	2 (2.5%)	
Normal Weight (18.5- 24.9)	108 (41.2%)	34 (42.5%)	
Overweight (25–29.9)	94 (35.9%)	26 (32.5%)	
Obese (> 40)	53 (20.2%)	19 (22.8%)	
Hb (g/dL) Anaemic	141 (53.8%)	46 (57.5%)	0.562
Ocular parameters, mean ± SD			
AC depth (mm)	3.67 ± 1.05	3.87 ± 1.01	**0.028**
Lens thickness	3.29 ± 0.89	3.30 ± 0.85	0.913
AA (cm)	2.46 ± 1.14	2.23 ± 1.24	0.092
NPA (cm)	48.15 ± 28.05	55.18 ± 51.77	0.651
NPC (cm)	9.5 ± 1.28	9.67 ± 1.18	0.229
Axial length (mm), n (%)			0.554
< 22	51 (19.5%)	18 (22.5%)	
22–26	211 (80.5)	62 (77.5%)	
Refractive error For distance			**0.021**
Emmetropes	171 (65.3%)	50 (62.5%)	
Myopes	29 (11.1%)	16 (20%)	
Hypermetropes	62 (23.7%)	14 (17.5%)	

BMI: Body mass index.

Hb: Haemoglobin.

AA: amplitude of accommodation.

NPA: near point of accommodation.

NPC: Near point of convergence.

Significant values are in [bold].

Those patients who had complaints related to presbyopia were further divided into 2 groups based on their willingness to accept presbyopic correction. Those with higher educational qualifications (*p* = 0.02) and skilled workers were more likely to accept near vision glasses (*p* = 0.02), while those with lower Hb (*p* = 0.01) and myopia (*p* = 0.01) were less likely to accept correction for presbyopia. (Tables 3 and 4).

**Table 3:** Comparison of socio-demographic parameters based on patient’s willingness to accept presbyopic correction.

Based on patient’s willingness to wear near vision (NV) glasses						
	Patients with complaints related to presbyopia, (n = 262)	*p* value		Patients without complaints related to presbyopia, (n = 80)	*p* value	
	Willing for NV correction	Not willing for NV correction		Willing for NV correction (n = 45)	Not willing for NV correction (n = 35)	
	(n = 240)	(n = 22)				
Age (years), mean ± SD	48.22 ± **6.57**	47.77** ± 6.67**	0.95	50.87** ± 6.5**	48.37** ± 7.35**	0.443
Gender						0.13
Male	112 (46.66%)	8 (36.36%)	0.35	22 (48.89%)	23 (65.71%)	
Female	128 (53.33%)	14 (63.64%)		23 (51.11%)	12(34.29%)	
Educational qualification			**0.02**			0.37
Illiterate	24 (10%)	4 (18.18%)		9 (20.00%)	7 (20.00%)	
Primary School	62 (25.83%)	10 (45.45%)		17 (37.78%)	11 (31.43%)	
Secondary School	70 (29.16%)	7 (31.82%)		14 (31.11%)	8 (22.86%)	
Above Graduate	84 (34.99%)	1 (4.55%)		5 (11.09%)	9 (25.71%)	
Occupation (Based on—classification)			**0.02**			0.07
Skilled	192 (79.99%)	12 (54.55%)		34 (75.55%)	20 (57.14%)	
Semiskilled	21 (8.75%)	4 (18.18%)		6 (13.33%)	4 (11.43%)	
Unskilled	27 (11.25%)	6 (27.27%)		5 (11.11%)	11 (31.43%)	

Significant values are in [bold].

**Table 4:** Comparison of ocular and systemic parameters parameters based on pateints' willingness to accept presbyopic correction.

	Patients with complaints of presbyopia (*n* = 262)	*P* value		Patients having no complaints of presbyopia (*n* = 80)	*p* value	
	Willing for NV correction (n = 240)	Not willing for NV correction (n = 22)		Willing for NV correction (n = 45)	Not willing for NV correction (n = 35)	
Systemic parameters n (%)						
Hypertensives	40 (16.66%)	3 (13.64%)	0.71	13 (28.89%)	11 (31.43%)	0.8
Diabetics	58 (24.16%)	9 (40.91%)	0.09	20 (44.44%)	12 (34.29%)	0.36
Patients taking chronic medications	69 (28.75%)	9 (40.91%)	0.23	24 (53.33%)	12 (34.29%)	0.09
BMI (kg/m2)			0.07			**0.04**
Under Weight	5 (2.08%)	1 (4.55%)		1 (2.22%)	2 (5.71%)	
Normal Weight	101 (42.08%)	7 (31.82%)		13 (28.89%)	20 (57.14%)	
Overweight	89 (37.08%)	5 (22.73%)		18 (40.00%)	8 (22.86%)	
Obese	45 (18.74%)	9 (40.90%)		13 (28.88%)	5 (14.28%)	
Hb(g/dL) anaemic	123 (51.25%)	18 (81.82%)	**0.01**	26 (57.78%)	20 (57.14%)	0.95
Ocular parameters (mean ± SD)						
AC depth (mm)	3.7 ± 1.06	3.56 ± 0.78	0.177	3.81 ± 0.96	3.94 ± 1.09	0.345
Lens thickness (mm)	3.31 ± 0.86	3.3 ± 0.80	0.788	3.33 ± 0.78	3.27 ± 0.95	0.151
AA (cm)	2.45 ± 1.13	2.66 ± 1.31	0.161	2.04 ± 1.08	2.49 ± 1.40	0.204
NPA (cm)	48.97 ± 28.84	39.18 ± 14.74	0.269	61.0 ± 62.23	47.61 ± 33.12	0.149
NPC (cm)	9.53 ± 1.28	9.18 ± 1.25	0.939	9.88 ± 1.18	9.4 ± 1.14	0.986
Axial length (mm),			0.33			0.94
n (%)						
< 22	45 (18.75%)	6 (27.27%)		10 (22.22%)	8 (22.86%)	
22–26	195 (81.25%)	16 (72.73%)		35 (77.78%)	27 (77.14%)	
Refractive error for distance			**0.01**			**0.05**
Emmetropes	163(67.91%)	8(36.6%)		25(55.6%)	25(71.4%)	
Myopes	24(10%)	5(22.73%)		8(17.8%)	8(22.9%)	
Hypermetropes	53(22.1%)	9(41%)		12(26.7%)	2(5.8%)	

Significant values are in [bold].

Of the 80 patients who did not present with chief complaints of presbyopia, 35 (43.75%) were not willing to accept near vision glasses. In this sub group, those with higher BMI (*p* = 0.04) and hypermetropes (*p* = 0.05) were more willing to accept presbyopic correction. (Tables 3 and 4).

Data Availability Statement—The datasets generated and/or analysed during the current study are available in the google drive repository—https://docs.google.com/spreadsheets/d/1y_9lIOfweSNzd22MjEPs-T4CoTyWtpAu/edit?usp=sharing&ouid=114933641242855917923&rtpof=true&sd=true

## Discussion

The present study noted over 3/4th of the patients above the age of 40 visiting the department of ophthalmology had complaints related to presbyopia. The observations of this study suggests that patients with higher educational qualifications, those with DM, HTN and requiring chronic medications, hypermetropia and lower AC depth were more likely to present with chief complaints of presbyopia. Those with higher educational qualification, professional or skilled workers were more likely to accept presbyopic correction, while myopes and those with anaemia were less likely to accept the same. Patients who came with complaints of presbyopia were more likely to accept near vision correction.

The present study observed a male: female (M:F) ratio of 0.8:1 among those who presented with complaints of presbyopia. When compared with M:F ratio of 1.3:1 among those above the age of 40 who did not have complaints of presbyopia, it appears that women are more likely to present with complaints of presbyopia. This was similar to Andhra Pradesh Eye Disease Study which observed female gender to have a greater association for presbyopia^1,11^. In addition, Priyambada S et al. and Mukuria M et al. also observed that women had earlier onset of presbyopia^1,12^.

Patients with higher education are more likely to be involved with near work, both in professional and nonprofessional spheres. They are therefore more likely to complain of presbyopia and because of the perceived need, also accept correction. Observations by Patel I et al. and Muhammad R et al. also noted that patients with higher education were more likely to be corrected for presbyopia^13,14^. Mukuria M et al. had a contrary observation of more severe presbyopia among those who were less literate. The authors explained it by suggesting a misinterpretation of the near vision charts, where those less literate may have preferred a magnified and therefore “clearer” correction^12^.

Association of education and occupation with a perceived need for presbyopic correction is a little blurred due to the increased use of digital media. Those whose occupations do not strictly require near vision, would perceive a need for presbyopic correction in other spheres. On the other hand, the ability to change the font size in digital screen permits postponement of presbyopic correction.

Skilled and professional jobs have a higher requirement of near vision acuity and therefore these patients may be more likely to accept presbyopic correction. Similar to our observations, a study in Nigeria also noted skilled professionals were more likely to procure glasses required for their near work^14^. In a study on unfulfilled need of presbyopic correction, Girum M et al. noted that those with higher education and skilled professionals were more likely to be corrected for their presbyopic needs earlier^15^. In parallel, a significant improvement in economical productivity has been observed following presbyopic correction even among those having unskilled jobs^8,16^. This emphasises the need for near vision correction irrespective of occupation.

Some studies have tried to determine the barriers for presbyopic correction. Hutchin B et al. in United Kingdom observed comfort and convenience of handling near vision glasses to be an important factor, more so than the cost of glasses. The perception that near vision glasses were a sign of aging also contributed to reluctance for presbyopic correction^3^. Contrary to this, in resource scarce countries, cost of glasses and ease of availability were noted to be significant barriers^8,14,16^. The observations in the present study that patients with skilled and professional jobs were more likely to accept presbyopic correction may have to do with need as well as affordability, though the latter was not expressly stated. Once corrected, the compliance towards near vision glasses was found to be above 80% even among unskilled labourers^16^. This suggests visual impairment due to presbyopia could be significantly addressed by economical support and access to eye care.

Despite chief complaints of presbyopia, some patients were unwilling to accept correction. Significant among these were patients with low Hb%. People with chronic illnesses like DM, HTN and those requiring chronic medications have the need to read and identify their medications in small prints. These patients may also present earlier with complaints of presbyopia due to compromised accommodation^17,18^. Surprisingly, we did not find a higher rate of acceptance for near vision correction among them. Systemic illness and priority for this over presbyopic correction may be a reason.

At the same time, participants with higher BMI (overweight and above) were more likely to accept near vision correction even when they did not have chief complains related to presbyopia.

Myopes were less likely to present with complains of presbyopia and also less willing to wear near vision glasses. Hypermetropes have lesser AA^19^. So they may present earlier and also perceive greater need for presbyopic correction. The observation that patients with lower AC depth were more likely to present with complaints related to presbyopia may be attributed to hypermetropic refraction in them^20^.

The present study highlights the fact that even among the patients who have access to health care facility uncorrected presbyopia is widely prevalent. Not many studies so far have explored patients’ need and willingness to accept presbyopic correction even though presbyopia is an important cause for visual impairment worldwide.

There were few limitations to this study. It was a single centre study. Studies involving multiple centres may be better able to address factors like socio-economic and geographical variations which would yield a deeper understanding into the needs and acceptance of presbyopic correction. Affordability for presbyopic glasses was inferred from education and profession and was not specifically studied. Further, grading of lens opacification was not done for participants. This could have affected NPA, thereby influencing their complaints related to presbyopia and decision for wearing glasses. Also, only patients’ willingness to accept presbyopic correction at the time of prescription was noted. Longitudinal study would be better to determine the patient compliance.

## Conclusion

Presbyopia is a significant cause for hospital visit in patients above the age of 40. Patients with higher educational qualifications, requiring chronic medications and hypermetropes were more likely to present with complaints of presbyopia. Those with higher education, skilled profession hypermetropes and higher BMI were more likely to accept presbyopic correction, while those with anaemia were less likely to accept near vision correction.

### Supplementary Information

Supplementary Information.
