# Author Correction: Factors influencing the need and willingness for presbyopic correction: a cross sectional study from south India

**DOI:** 10.1038/s41598-024-52891-x

**Published:** 2024-01-30

**Authors:** Dhruval A. Khurana, N. Swathi, A. R. Rajalakshmi

**Affiliations:** Department of Ophthalmology, Mahatma Gandhi Medical College and Research Institute, Sri Balaji Vidyapeeth, Pondicherry, India

Correction to: *Scientific Reports* 10.1038/s41598-023-50288-w, published online 21 December 2023

In the original version of this Article, Dhruval A. Khurana was incorrectly listed as a corresponding author. The correct corresponding author for this Article is N. Swathi. Correspondence and request for materials should be addressed to swats1309@yahoo.com.

The original Article has been corrected.

